# Supplementary figures and images for: Deubiquitinating activity of SARS-CoV-2 papain-like protease does not influence virus replication or innate immune responses in vivo
Source: PLoS Pathog. 2024 Mar 25;20(3):e1012100. doi: 10.1371/journal.ppat.1012100 (PMC10994560; doi:10.1371/journal.ppat.1012100)

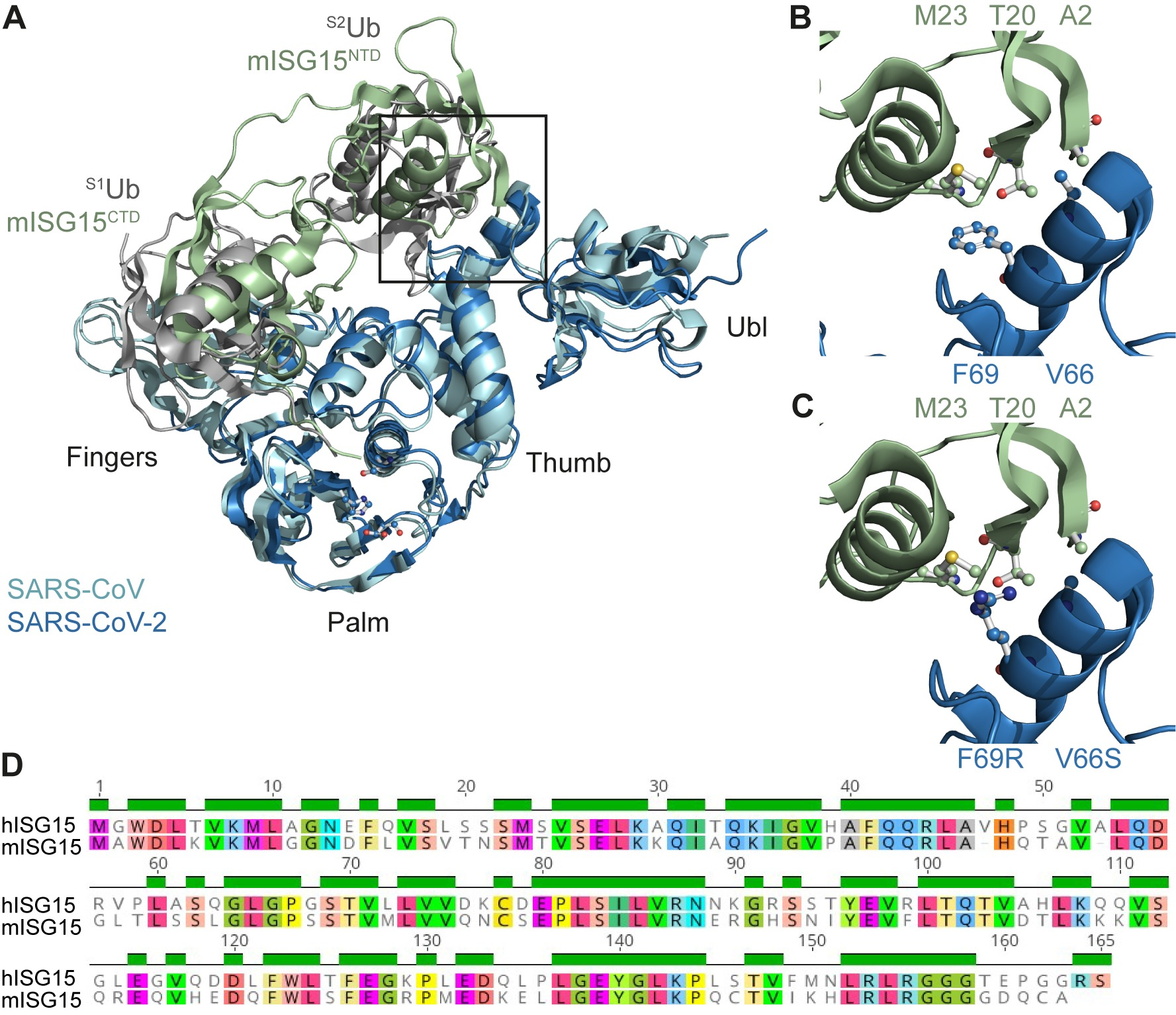

Supplement: S1 Fig — (A) Cartoon representation of SARS-CoV PLpro (aquamarine) in complex with K48-linked diubiquitin (grey; PDB 5E6J) aligned with SARS-CoV-2 PLpro (sky blue) in complex with mouse ISG15 (pale green; PDB 6YVA). Catalytic triad residues (C111, H272, D286) are shown in ball-and-stick representation. (B-C) Interaction of PLpro residues V66 and F69 with mISG15 residues A2, T20, and M23 (B), which is predicted to be disrupted by introduction of V66A and F69R (C). (D) Alignment of human (UniProt P05161) and mouse (UniProt Q64339) ISG15. Mouse ISG15 residues A2 and T20 are not conserved. (TIF) [file ppat.1012100.s001.tif]

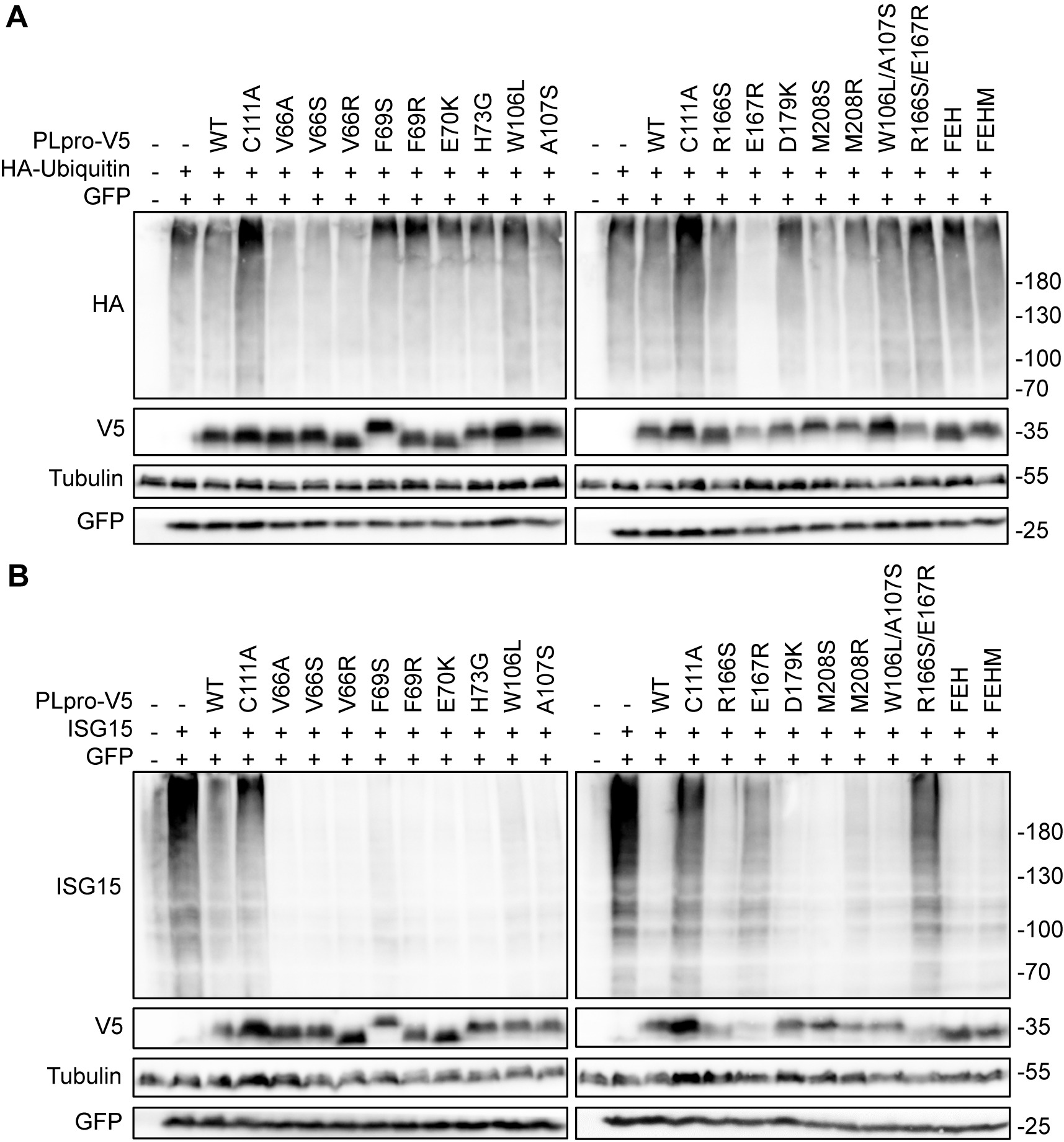

Supplement: S2 Fig — (A) Immunoblot analysis of ubiquitin conjugation in HEK293T cells transfected with ubiquitin and SARS-CoV-2 PLpro wild-type or mutants at 24 hours post transfection. (B) Immunoblot analysis of ISG15 conjugation in HEK293T cells transfected with ISG15 and SARS-CoV-2 PLpro wild-type or mutants at 48 hours post transfection. FEH(M) = F69S/E70K/H73G(/M208S). Representative blots of n = 5 experiments. (TIF) [file ppat.1012100.s002.tif]

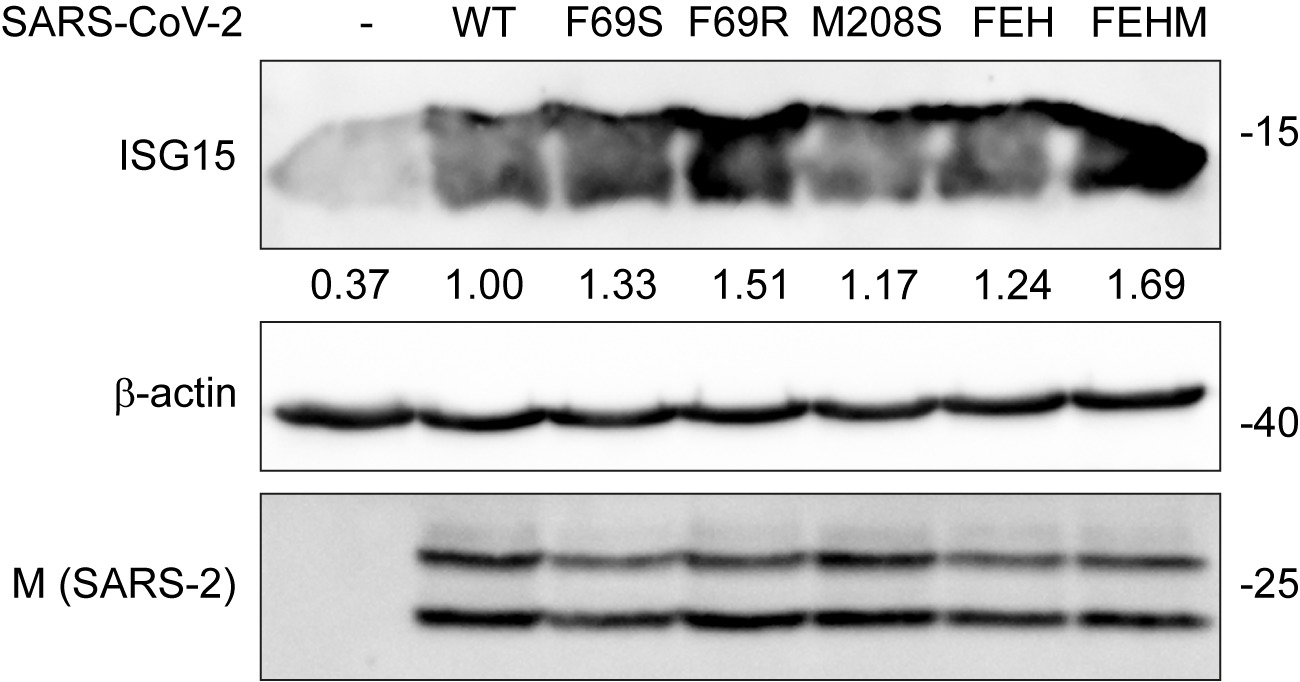

Supplement: S3 Fig — Calu-3 cells were infected at MOI 0.1 with wild-type or DUB mutant SARS-CoV-2 and protein lysates were collected at 24 hpi to analyze ISG15 protein expression by western blot. ISG15 expression was normalized to β-actin expression and fold changes were calculated relative to wild-type SARS-CoV-2. Representative blots of n = 2 experiments. FEH(M) = F69S/E70K/H73G(/M208S). (TIF) [file ppat.1012100.s003.tif]

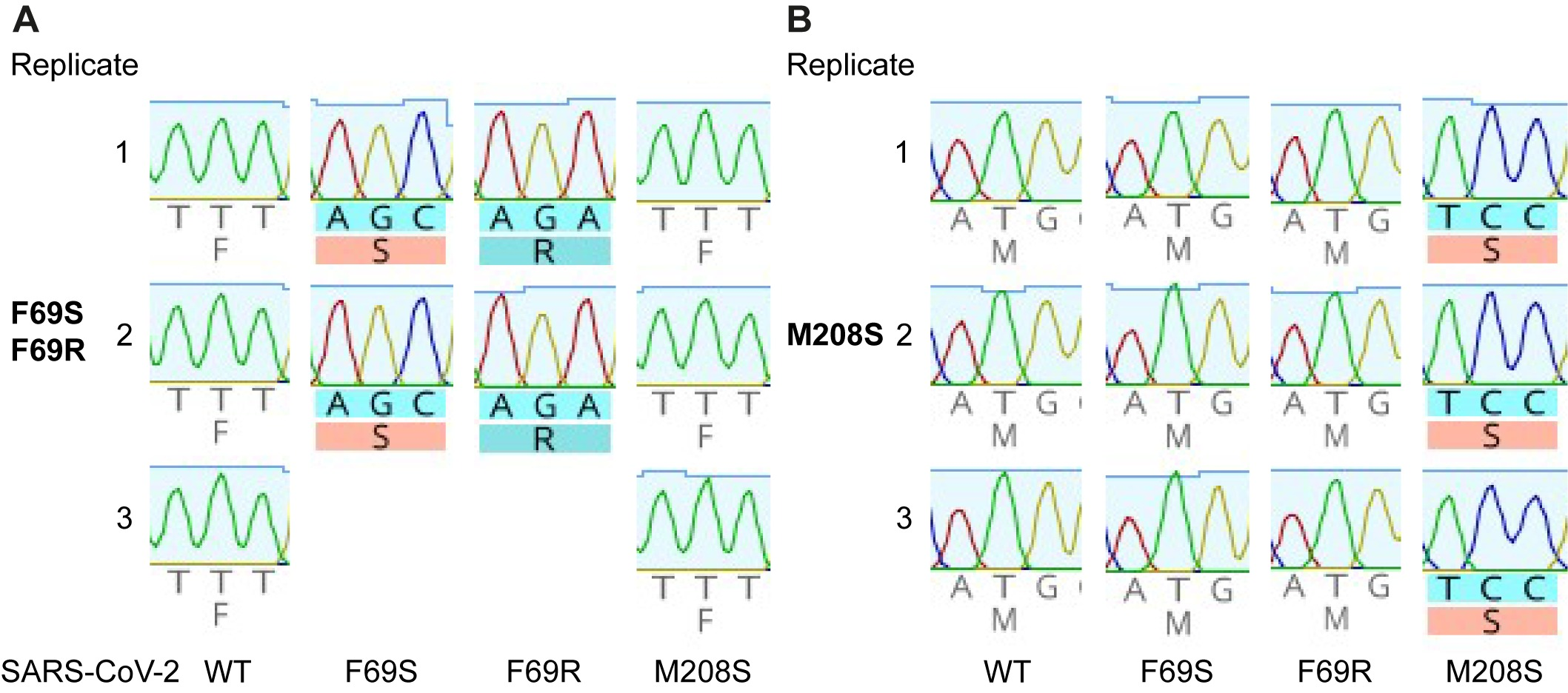

Supplement: S4 Fig — (A-B) Genetic stability of F69S and F69R (A) and M208S (B) in vivo was determined by RT-PCR to amplify the PLpro-coding region from RNA extracted from lung homogenates harvested at 4 dpi from infected mice. The PCR product was Sanger sequenced. 2–3 mice were analyzed per virus. (TIF) [file ppat.1012100.s004.tif]

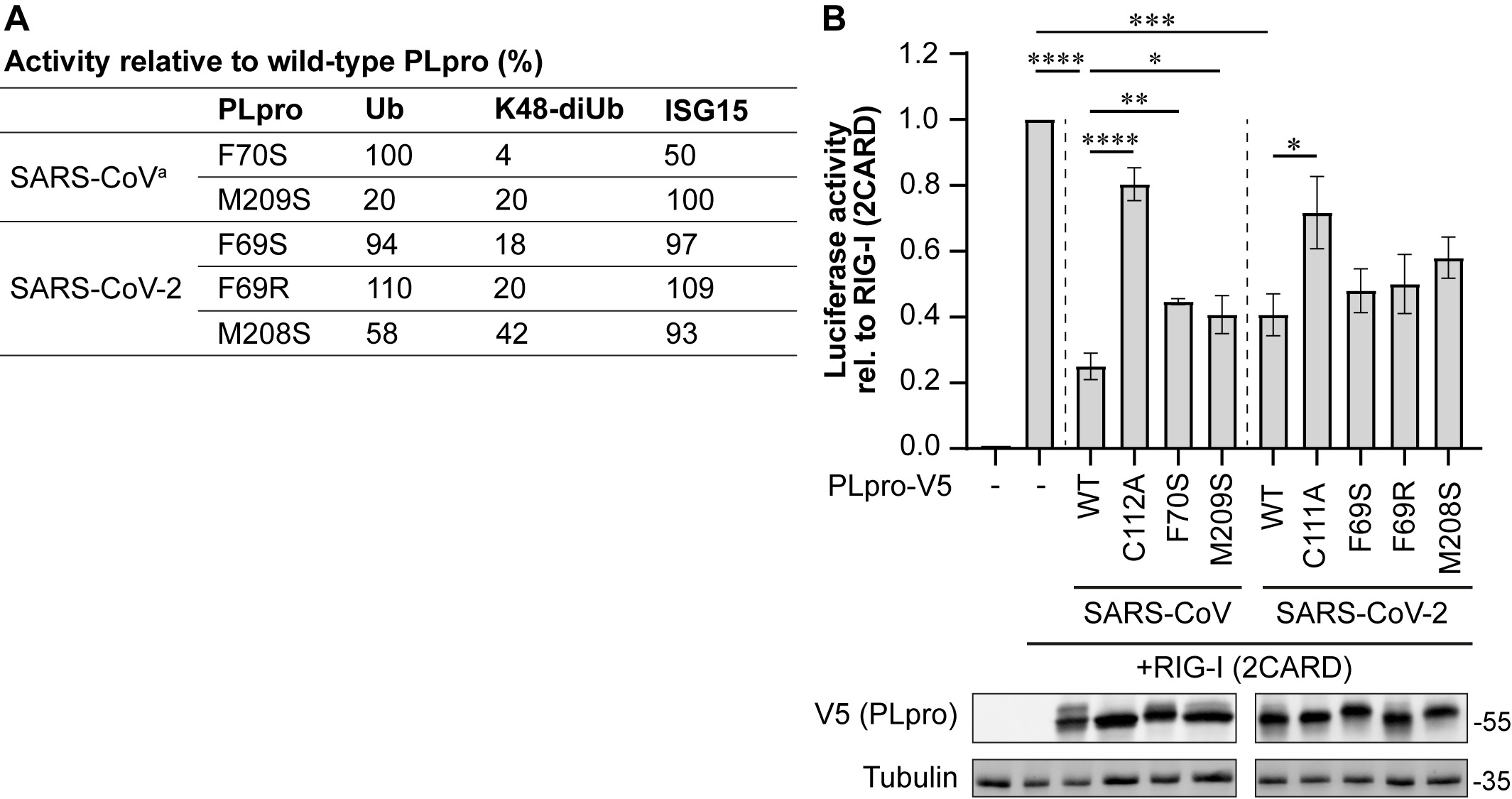

Supplement: S5 Fig — (A) Relative DUB and deISGylating activity of SARS-CoV and SARS-CoV-2 PLpro mutants compared to wild-type PLpro as assessed by the hydrolysis of Ub-AMC, K48-diUb-AMC, and ISG15-AMC. (B) IFNβ promoter-driven luciferase activity in HEK293T cells transfected with Renilla and firefly luciferase, wild-type or mutant SARS-CoV or SARS-CoV-2 PLpro, and constitutively active RIG-I2CARD. Luciferase activity was determined at 24 hours post transfection. Firefly luciferase was normalized to Renilla luciferase and expressed relative to the positive control, RIG-I2CARD. Panels below show representative immunoblots using V5 to verify expression of PLpro and α-tubulin as loading control. a Data in panel A for SARS-CoV PLpro is from Békés et al. [31]. Data for SARS-CoV-2 PLpro was taken from Fig 2. Data in B are represented as mean ± s.e.m. of 3 experiments. One-way ANOVA with Dunnett’s multiple comparisons test, comparing each group to wild-type PLpro. * p<0.05, ** p<0.01, *** p<0.001, **** p<0.0001. (TIF) [file ppat.1012100.s005.tif]

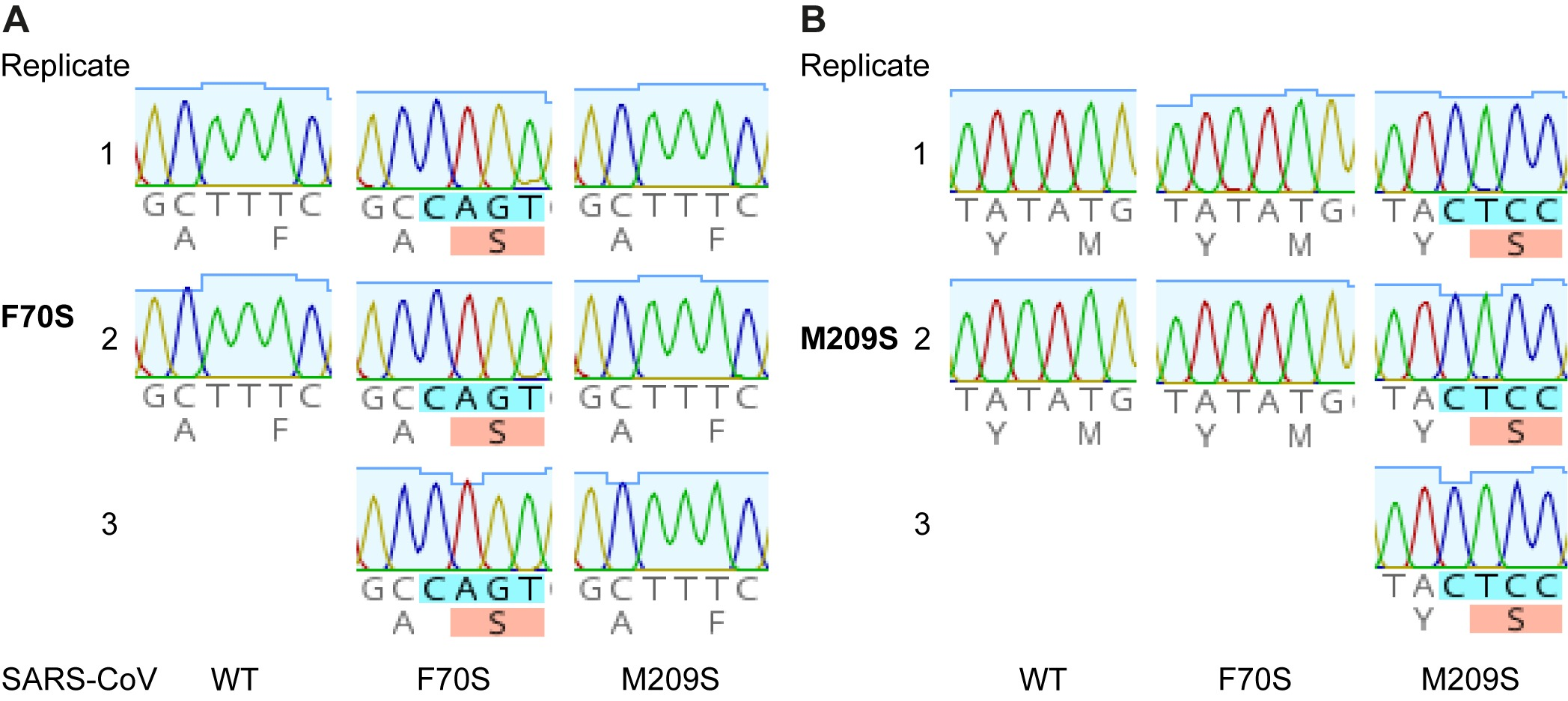

Supplement: S6 Fig — (A-B) Genetic stability of F70S (A) and M209S (B) in vivo was determined by RT-PCR to amplify the PLpro-coding region from RNA extracted from lung homogenates harvested at 4 dpi from infected mice. The PCR product was Sanger sequenced. 2–3 mice were analyzed per virus. Silent mutations in A69 and Y208 are marker mutations that were introduced on purpose to exclude contamination with the parental virus. (TIF) [file ppat.1012100.s006.tif]

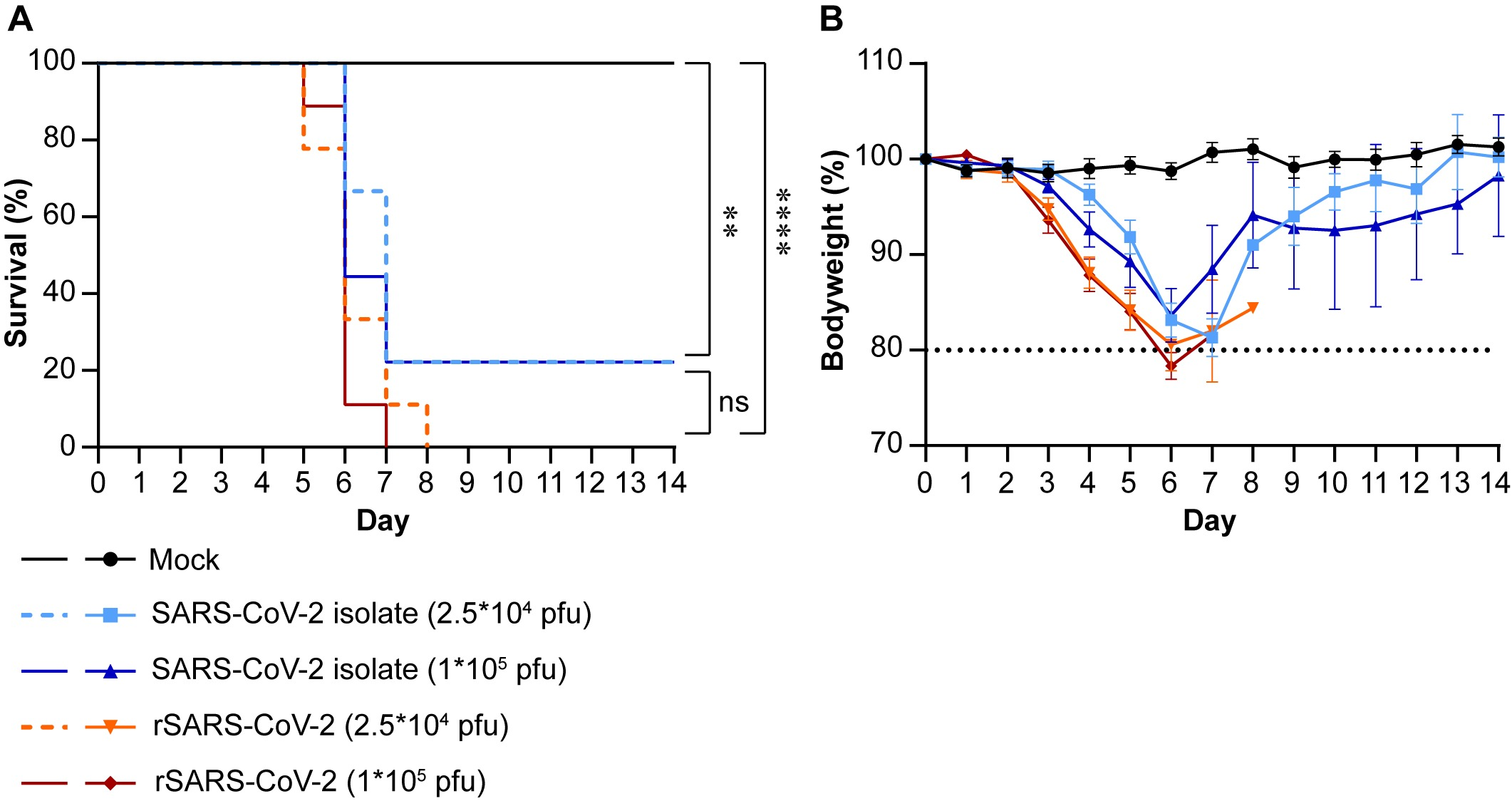

Supplement: S7 Fig — K18-hACE2 mice were infected intranasally with 2.5x10^4^ or 1x10^5^ pfu rSARS-CoV-2 or SARS-CoV-2 (isolate) or mock-infected with DMEM. (A) Survival curves (%). (B) Bodyweight loss (% from initial weight). Dashed line indicates 20% weight loss upon which mice are euthanized. n = 8 mice per group for mock and n = 9 for SARS-CoV-2-infected groups. Log-rank test for survival analysis (A). One-way ANOVA with Šídák’s multiple comparisons test (B). ns: not significant, *p<0.05, ** p<0.01, *** p<0.001, **** p<0.0001. (TIF) [file ppat.1012100.s007.tif]
