# Supplementary material for: Deubiquitinating activity of SARS-CoV-2 papain-like protease does not influence virus replication or innate immune responses in vivo
Source: PLoS Pathog. 2024 Mar 25;20(3):e1012100. doi: 10.1371/journal.ppat.1012100 (PMC10994560; doi:10.1371/journal.ppat.1012100)
Supplement: Attachment — Submitted filename: 20240229_Response_to_reviewers.docx [file ppat.1012100.s012.docx]

Leiden, 29 February 2024

Dear Dr. Frieman,

Herewith we submit our revised manuscript entitled “Deubiquitinating activity of SARS-CoV-2 papain-like protease does not influence virus replication or innate immune responses *in vivo*”.

We are grateful for the time and effort of the reviewers. We have revised our manuscript based on their input. Please find a point-to-point response to the reviewer’s comments below (in blue text). We have also uploaded the revised manuscript with tracked changes and a clean version. If changes were made in the manuscript, lines and page numbers mentioned refer to the revised version with tracked changes.

We hope that this revised version will be now suitable for publication in PLOS Pathogens.

Looking forward to hearing from you soon,

Kind regards,

Mariska van Huizen

**Part I – Summary**

Please use this section to discuss strengths/weaknesses of study, novelty/significance, general execution and scholarship.

Reviewer #1: In this study the authors aimed to determine the contribution of the DUB and deISGylating activities of the SARS-CoV-2 papain-like protease to viral replication and modulation of the innate immune responses. Using a structure-guided approach, they introduced several mutations in PLpro that specifically disrupt binding to ubiquitin or ISG15 without affecting the protease activity that processes the viral polyprotein. Biochemical assays confirmed reduced DUB and/or deISGylase activity for several viable mutants. By generating recombinant SARS-CoV-2 and SARS-CoV carrying representative PLpro DUB mutations, the authors provide evidence that the DUB function is dispensable for SARS-CoV-2 replication and disease in mice, while it plays a subtle role for SARS-CoV.

General comments:

This is well-conducted study using using both in vitro and in vivo approaches to dissect the multifunctional properties of PLpro during infection. The combined use of virological, immunological and animal experiments significantly extends our understanding about the differential roles of coronavirus PLpro domains as well as key differences between highly pathogenic family members.

We are happy that the reviewer considers the study to be well-conducted and appreciates the insights that this study provides.

Reviewer #2: The manuscript by van Huizen and colleagues describes the analysis of the SARS-CoV-2 DUB and deISGylating activities residing in PLpro. Since PLpro is essential for CoV polyprotein processing the aim was to un-couple DUB, deISGylation from the essential PLpro activity and to study the impact of DUB and deISGylation in the context of virus infection. Structure-guided aa changes in PLpro showed for some substitutions a lack or a reduced DUB or deISGylation activities, while for some changes PLpro poly processing activity remained intact. However, the impact of these changes on viral replication in vitro and in vivo remains elusive since no major impact could be identified. This is in stark contrast to a similar approach that was used for MERS-CoV where clear impact on virus replication, particularly in vivo has been observed.

Overall, this is very good and comprehensive study that will stimulate the field. The authors appropriately discussed possible reasons why DUB or deISGylation has only minor impact on SARS-CoV-2 replication compared to MERS-CoV.

We thank the reviewer for the positive words and appreciation for our study.

**Part II – Major Issues: Key Experiments Required for Acceptance**

Please use this section to detail the key new experiments or modifications of existing experiments that should be absolutely required to validate study conclusions.

Generally, there should be no more than 3 such required experiments or major modifications for a "Major Revision" recommendation. If more than 3 experiments are necessary to validate the study conclusions, then you are encouraged to recommend "Reject".

Reviewer #1: Specific comments:

In Figure 3B, its strange that infectious virus particles can be detected at 8 hours post infection. The SARS-CoV-2 life-cycle takes atleast 12 hours for a single round, which would indicate that what is being detected here might be the inoculum. Along the same lines, in Figure 3A, the no of plaques appear to be significantly lower with the mutant viruses compared to the wild-type virus, yet the pfu/ml values seem to be similar in Figure 3B. Are the dilutions shown in Figure 3A different in the mutants v/s the wild-type?

After the infection, cells are washed three times with PBS and the last wash is collected. We took this wash along in plaque assays and did not find any plaques in the -1 dilution, indicating that the amount of residual virus is less than 40 pfu/ml. We thus believe that the virus that is detected at 8 hours post infection is produced by the cells and is not the result of improperly removed inoculum. We have added a dotted line to the graph to show the limit of detection and have included the results from the wash. Furthermore, adjustments were made to the legend of Figure 3 in the revised manuscript to better explain the data shown (lines 1246-1253). The revised figure is depicted below.

Fig. 3A contains examples from plaque assays used for the titration of virus stocks. Stocks were serially diluted in 10-fold steps and plaqued on VeroE6 cells, since it is in our hands not possible to titrate on Calu-3 cells. Besides the serial dilution, no adjustments were made to the amount of input virus, which explains the difference in number of plaques. Based on the stock titers, inoculums were prepared for experiments presented in figure 3B, so that MOI 0.1 was used for all infections.

In Figure 4, it would be useful to see the immune responses measured at protein levels rather than measured solely at RNA levels. The authors should therefore measure cytokines and chemokines in cell culture supernatants and mouse lung homogenates by ELISA or multiplex immunoassay. Similarly, activation of cytokine signalling pathways such as STAT1/STAT3 phosphorylation might provide better indication of whether such signalling cascades are affected.

We agree with the reviewer that it would also be interesting to measure protein levels of the cytokines of interest. We measured IFNβ and IL-6 levels in the supernatants of infected Calu-3 cells at 24 and 48 hours post infection. IFNβ was only detected at 48 hpi. See below the data from three independent experiments. The amount of IFNβ and IL-6 that was produced by the cells varied a lot from experiment to experiment. Unfortunately, we were not able to identify consistent differences that provided statistical significance. Therefore, we focused on the RNA expression of IFNB and several ISGs and cytokines and repeated those experiments at least 5 times, to verify that the differences that are observed are reproducible. Because of the variation between experiments in the protein data, we have not included this data in the manuscript.

In mice we already observed a lot of heterogeneity in cytokine expression at the RNA level. We expect that this would be even more pronounced at the protein level. However, we have now measured IL-6 in the lungs of mice, since IL-6 is one of the cytokines that peaks rather early upon infection. The data is shown below. We did not find a difference in IL-6 levels between the mock and infected mice at 2 days post infection. At 4 days, IL-6 levels seem to decrease slightly in the SARS-CoV-infected mice as compared to the mock. Since there is no clear induction of IL-6 in the lungs of the infected mice, it is hard to draw any conclusions on the effects of the introduced mutations. One would probably have to analyze later timepoints and we originally planned to also collect organs at 6 dpi. However, we could not do this since some mice already had to be sacrificed at 5 dpi (Fig. 5A and 7A). We have therefore not included this data in the manuscript.

The reviewer also suggested to analyze the activation of interferon signaling by western blot. We have performed western blot for ISG15, both in its unconjugated and conjugated form. MonoISG15 was detected very well. Below we depict a representative example of two independent experiments. ISG15 expression was normalized to β-actin levels and fold change was calculated relative to SARS-CoV-2 WT. ISG15 levels are increased by SARS-CoV-2 infection. The DUB mutants induce more ISG15 expression than the wild type and this is in line with the trend that was observed in the RNA levels at 12 and 24 hpi (Fig. 4B-C, ISG15 panels inserted on the right for reference). This data has now been added to the manuscript as Figure S3. We could hardly detect conjugated ISG15, so we were not able to evaluate the levels of ISG15 conjugation upon infection.

Fig. S3	Fig. 4B	Fig. 4C

For the mice infection studies (Figure 6 and 7), while the PLpro mutant viruses display modest defects in replication, combination with other attenuating mutations may reveal synergistic processes, which in turn might show differences in cytokine profiles. Similarly, larger group sizes may be needed to detect significance in replication differences.

As the reviewer points out, it would be very interesting to combine the PLpro mutations with other attenuating mutations. We have also suggested this in the discussion as a subject for future studies (lines 499-516).

The reviewer points out that with larger group sizes, we might be able to detect significant differences in virus replication. While planning the animal experiments, power analysis was performed to calculate the required group size prior to detect significant differences. Based on this, group size was set to 4 mice for the mock-infected controls and to 6 mice for the groups infected with SARS-CoV-2 or SARS-CoV. It could be that with a larger group, the differences that are now borderline significant, would actually become statistically significant. However, the actual effect size will not increase. From an ethical point of view, our local animal welfare body would not approve the use of more animals for such small differences. Future studies beyond the scope of this work would need to investigate the combination of our PLpro mutations with other attenuating mutations as discussed in lines 499-516 in the revised manuscript with track changes.

Besides cytokine responses, it would be useful if the authors could also evaluate and include antibody titres in mouse serum by ELISA at various time points post-infection and CD4+ and CD8+ T cells in lungs and spleen. This would provide greater insights into the role of PLpro’s DUB activity in functional properties and kinetics of adaptive immune responses during SARS-CoV-2 infection and disease pathogenesis.

We agree with the reviewer that it would be valuable to gain more insight into the effects of the removal of PLpro’s DUB activity on adaptive immune responses. However, this would require additional mouse experiments of longer duration to allow analysis of adaptive responses in surviving mice, and we consider this to be beyond the scope of this study.

Reviewer #2: no major concerns

**Part III – Minor Issues: Editorial and Data Presentation Modifications**

Please use this section for editorial suggestions as well as relatively minor modifications of existing data that would enhance clarity.

Reviewer #1: Some of the figure legends do not contain adequate experimental details.

We apologize for this and have now added some experimental details, such as the MOI at which the cells were infected, to the legends of figures 3 and 4.

Reviewer #2: 1. Results section, biochemical characterisation (lines 179ff): Please add a few sentences to introduce the experimental setup. It is unclear for the reader at first glance (without digging into the methods section) how PLpro was expressed and used for the assays.

We thank the reviewer for this request. We changed the first lines of this paragraph to better explain the experimental set-up of the biochemical characterization. We hope that this provides extra clarity for the reader.

2. Figure 1A: A simple illustration of PLpro in relation to the viral genome and depiction of introduced changes would be good to have in addition to the structure.

We thank the reviewer for his/her suggestion and we have now added a schematic in figure 1B to show the location of the PLpro domain in the viral polyprotein, the nsp junctions that are cleaved by PLpro, and the interaction with ubiquitin and ISG15. Black asterisks indicate the positions where mutations were introduced to disrupt binding to ubiquitin and/or ISG15. Orange asterisks indicate position of catalytic residues. The revised version of the figure is depicted below.

3. In vivo experiments. While the authors did not see major differences in viral load (RNA and titers) and only minor differences in some cases for host responses, it might be interesting to include pathology. In some cases (Fig 5B) recovery from weight loss is delayed compared to wt. Together with the observation that some cytokines are slightly increased for some mutants, it might be good to check if this may impact disease/lung pathology. If those data are available it would be good to add them.

The reviewer is right in pointing out that in the case of SARS-CoV-2, there seems to be a difference in recovery from weight loss. However, it is important to take into account that each group contains only 1-3 mice at those days and we should be careful drawing conclusions from such small groups. Since we did not observe a difference in survival, viral load, or immune responses in the case of SARS-CoV-2, we considered it highly unlikely that there would be major differences in lung pathology. Therefore, the cost and effort of pathology analysis could not be justified and we can unfortunately not provide this data.

4. Is it possible to check for DUB or deISGylation activity of mutant viruses versus wt virus in infected cells?

We agree with the reviewer that it would be very interesting to check for the DUB or deISGylation activities of the mutant viruses versus wt virus in infected cells. We tried to assess ubiquitin and ISG15 smears in infected cells, but did not manage to get consistent results. MonoISG15 could be detected very well, but we could hardly detect ISGylated proteins. MonoISG15 protein levels were increased upon infection and the DUB mutant viruses induced higher expression than the wild-type virus. We have now included the western blot data for monoISG15 as Fig. S3. Ubiquitin smears can be detected very well. However, the wild-type virus only gives a minor reduction in conjugated ubiquitin levels, most likely due to its specificity for K48-linked ubiquitin chains. As the majority of ubiquitin conjugates in cells are monoubiquitinated substrates, there is a lot of background in the ubiquitin smears. This made it hard to show consistent differences between the wild-type and the DUB mutants. This could potentially be solved by the use of a K48-linked ubiquitin chain specific antibody. However, these antibodies are mainly used on overexpressed ubiquitin or to detect ubiquitination on immunoprecipitated substrates. The signal for endogenous K48-linked ubiquitin chains is very low to non-detectable. This could be increased by the addition of proteasome inhibitors, but these inhibit SARS-CoV-2 infection and could therefore not be used in our experiments. All-in-all we therefore decided to analyse DUB and ISGylation activity of mutant PLpro proteins using *in vitro* assays as presented in the paper.
